# Organic Black Beans (*Phaseolus vulgaris* L.) from Rio de Janeiro State, Brazil, Present More Phenolic Compounds and Better Nutritional Profile Than Nonorganic

**DOI:** 10.3390/foods10040900

**Published:** 2021-04-19

**Authors:** Nathália M. B. Barreto, Natália G. Pimenta, Bernardo F. Braz, Aline S. Freire, Ricardo E. Santelli, Angélica C. Oliveira, Lucia H. P. Bastos, Maria Helena W. M. Cardoso, Mariana Monteiro, Maria Eduarda L. Diogenes, Daniel Perrone

**Affiliations:** 1 Laboratory of Nutritional Biochemistry and Food, Chemistry Institute, Federal University of Rio de Janeiro, 149 Av. Athos da Silveira Ramos, CT, Bloco A, sala 528A, Rio de Janeiro 21941-909, Brazil; nathaliambb.nut@gmail.com (N.M.B.B.); nataliagpimenta@gmail.com (N.G.P.); 2 Laboratory of Functional Foods, Nutrition Institute, Federal University of Rio de Janeiro, 373 Av. Carlos Chagas Filho, CCS, Bloco J, 2 andar, sala 16, Rio de Janeiro 21941-902, Brazil; mariana@nutricao.ufrj.br; 3 Technical Area of Food, Nutrition, Physical Activity and Cancer, National Institute of Cancer, Ministry of Health, 125 Rua Marquês de Pombal 5 andar, Rio de Janeiro 20230-240, Brazil; maria.melo@inca.gov.br; 4 Laboratory of Analytical Development, Chemistry Institute, Federal University of Rio de Janeiro, 149 Av. Athos da Silveira Ramos, CT, Bloco A, sala 518A, Rio de Janeiro 21941-909, Brazil; bernardobraz@id.uff.br (B.F.B.); alinesoaresfreire@gmail.com (A.S.F.); santelli@iq.ufrj.br (R.E.S.); 5 Laboratory of Pesticide Residues, Chemistry Department, National Institute of Quality Control in Health (INCQS), Oswaldo Cruz Foundation (FIOCRUZ), 4365 Av. Brasil, Rio de Janeiro 21045-900, Brazil; angelica.oliveira@incqs.fiocruz.br (A.C.O.); lucia.bastos@incqs.fiocruz.br (L.H.P.B.); helena.wohlers@incqs.fiocruz.br (M.H.W.M.C.); 6 Department of Basic and Experimental Nutrition, Nutrition Institute, State University of Rio de Janeiro, 524 Rua São Francisco Xavier, Pavilhão João Lyra Filho, 12º andar, Bloco D, sala 12.023, Rio de Janeiro 20559-900, Brazil

**Keywords:** minerals, pesticides, phenolic compounds, phytate, production system, protein

## Abstract

Brazil is the world’s third largest common bean (*Phaseolus vulgaris* L.) producer, and 60% of its population consumes this legume. Although organic farming is a sustainable alternative to nonorganic agriculture, its effect on chemical composition is still controversial. Therefore, the aim of this study was to investigate differences in the nutritional and phenolic compounds profiles between organically and nonorganically produced Brazilian black beans. Samples were obtained from the same harvest periods and from near geographical locations at metropolitan and coastal regions of Rio de Janeiro state, Brazil. No residues of 294 evaluated pesticides were detected in the samples. In both regions, organic beans had 17% fewer lipids, 10% less phytate and 20% more proteins when compared to nonorganic ones. Sixteen different phenolic compounds were identified as soluble and insoluble forms in black beans, with anthocyanins being the most abundant (on average, 66%). In both regions, soluble and total phenolic compounds contents in organic beans were consistently higher (on average, 25% and 28%, respectively) than in nonorganic ones. Our results show that organic farming improves the nutritional profile and increases the phenolic compounds content of black beans.

## 1. Introduction

Brazil is the world’s third largest dry bean producer, behind Myanmar and India, with a production corresponding to approximately 10% of the 28.9 million tons produced in 2019, according to FAO [1]. Although consumption of this legume has been decreasing over time, beans are typical of Brazilian cuisine and, according to the latest available data, consumption frequency by the general population is 60%. Moreover, this legume is one of the most consumed foods in Brazil, with a daily average of 142 g per capita [2].

In general, legumes present high levels of protein, fiber, minerals and complex carbohydrates, in addition to having low levels of lipids [3]. Among minor compounds, legumes show phenolic compounds, as well as phytates and trypsin inhibitors, which are considered antinutritional factors [4]. Studies show that legumes have antioxidant activity both in vitro and in animals [5,6]. Particularly for beans, several studies have reported that their consumption is associated with a lower risk of developing noncommunicable diseases, such as some types of cancer, cardiovascular diseases, diabetes mellitus, and obesity [7]. These benefits are not only associated with the presence of fibers, proteins, minerals and vitamins, but also with bioactive compounds, mainly phenolic compounds [8,9]. In vitro studies that evaluated bean extracts rich in phenolic compounds have observed both antiproliferative and anti-inflammatory effects [10,11,12].

Beans are an important dietary source of proteins, minerals and dietary fibers, and are rich in flavonols and anthocyanins [13]. In fact, beans are the second most important dietary source of phenolic compounds in Brazil [14]. In general, the nutritional and bioactive compounds profile of foods may be influenced by climate conditions, soil, and cultivar, as well as agricultural practice (i.e., nonorganic or organic farming) [15,16]. Since phenolic compounds are secondary plant metabolites, their biosynthesis is especially affected by stresses during cultivation, which is closely related to agricultural practice [17].

Organic farming has been recognized as a sustainable alternative to nonorganic agriculture. In addition to respecting social and cultural aspects, organic agriculture adopts sustainable practices throughout the production process, which is also characterized by not using pesticides and synthetic fertilizers. Consumers perceive organic foods as healthier and safer than nonorganic [18], which is reflected in the growing number of organic producers throughout the world. However, there are only few studies about the health effects of organic food consumption. The Nutrinet-Santé cohort study reported that, among French adults, those with higher frequency of organic food consumption had a lower risk of cancer, probably due to lower ingestion of pesticides by the organic food consumers [19]. Moreover, studies that evaluated the effect of organic farming on food chemical composition showed controversial results. While some authors reported small or no differences in minerals [15] and phenolic compounds contents between organic and nonorganic food [15,20], in a meta-analysis study, Barański et al. [16] observed lower levels of protein and fibers and higher levels of phenolic compounds in organic foods in comparison to nonorganic. Furthermore, there are a few studies evaluating differences between production systems, especially in relation to legumes.

In this way, the objective of this study was to investigate differences in the nutritional and phenolic compound profiles between organically and nonorganically produced black beans.

## 2. Materials and Methods

### 2.1. Chemicals and Materials

Standard solutions of minerals were purchased from Quimlab Química & Metrologia^®^ (São Paulo, Brazil). Ion exchange column AG^®^ 1-X8 was purchased from BIO RAD (Hercules, CA, USA). Kjeldahl catalyst was purchased from Vetec (Rio de Janeiro, Brazil). Suprapur^®^ sodium acetate, EMSURE anhydrous magnesium sulphate and formic acid were obtained from MERCK^®^ (Darmstadt, Germany). Anthocyanin and non-anthocyanin standards were purchased respectively from Indofine Chemical Co. (Hillsborough, NJ, USA) and Sigma-Aldrich Chemical Co. (St. Louis, MO, USA and Milwaukee, WI, USA). The reference materials for pesticides were purchased from AccuStandard (New Haven, CT, USA) and Dr. Ehrenstorfer (Augsburg, Germany). Phytic acid was purchased from Aldrich Chemical Company, Inc. (Milwaukee, WI, USA). Total Dietary Fiber Assay Kit was purchase from Sigma-Aldrich (St. Louis, MO, USA). All solvents were High Performance Liquid Chromatography (HPLC) grade from Tedia (Fairfield, OH, USA) or MERCK^®^ (Darmstadt, Germany). HPLC grade water (Milli-Q System, Millipore, Bedford, MA, USA) was used throughout the experiments.

### 2.2. Black Bean and Soil Samples

**Figure 1:**
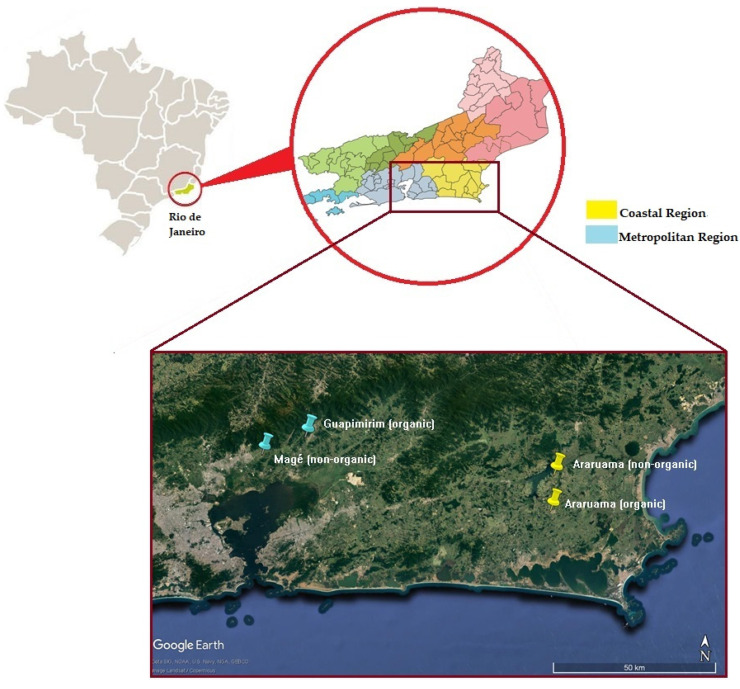
Map showing the coastal and metropolitan regions of Rio de Janeiro state, Brazil, where black bean samples were collected.

Four black beans (*Phaseolus vulgaris* L., cv. “BR1-XODÓ”) samples were obtained from two distinct regions of Rio de Janeiro state, Brazil. The first set of samples was from the metropolitan region: organic beans were grown at Guapimirim county (~22°33′33.6″ S, 43°00′24.1″ W) and the nonorganic ones at Magé county (~22°36′43.0″ S, 43°07′54.8″ W). The second set of samples was from the coastal region: both organic and nonorganic beans were grown at Araruama county (~22°43′21.9″ S, 42°15′57.8″ W and ~22°37′29.3″ S, 42°15′43.1″ W for organic and nonorganic beans, respectively). The distance between farms where organic and nonorganic beans were grown in the metropolitan and coastal regions was about 14 km and 10.5 km, respectively (Figure 1). Although the state of Rio de Janeiro is not a large producer of beans nationwide, it was chosen because there are organic and nonorganic producers located close to each other in both regions studied. All samples were harvested in the winter season, between July 1st and September 15th, 2016. Together, the chosen harvest locations and periods ensured that eventual chemical composition differences between samples could be associated with organic or nonorganic farmers rather than other effects, such as climatic conditions. Samples were ground in a laboratory mill and kept at −20 °C until analysis. 

Organic farms were certified through the Participatory Guarantee System (PGS) by the Association of Biological Producers of Rio de Janeiro (Associação de Produtores Biológicos do Estado do Rio de Janeiro—ABIO), which is accredited by the Brazilian Ministry of Agriculture, Livestock and Food Supply. It is important to describe that the coastal organic producer has been using a rhizobia bacteria-based inoculant in bean crops since 2013, in collaboration with Empresa Brasileira de Pesquisa Agropecuária (EMBRAPA) Agrobiologia.

Samples of soils in which the beans were grown were collected close to the harvesting period. They were ground, dried in an oven at 60 °C for 24 h, and kept at room temperature until analysis.

### 2.3. Soil Chemical Analysis

#### 2.3.1. Mineral Composition

Soil digestion was performed in duplicate according to United States Environmental Protection Agency (USEPA 3050b method) [21]. Calcium, phosphorus, iron, magnesium, manganese, potassium, copper, zinc and sodium contents in soil samples were determined using an optical emission spectrometer with inductively coupled plasma source (ICP OES), with radial vision (Horiba Jobin Yvon, Ultima 2, Longjumeau, France), equipped with a cyclonic spray chamber and a parallel flow nebulizer MiraMist type (Mira Mist EC, Burgener Research Inc., Ontario, Canada), AS 421 automatic sampler and Analyst 5.4 operational software for data acquisition. The quantification was performed by interpolation using analytical curve with four standard solutions for calibration. These solutions were prepared by diluting a standard stock solution SpecSol 1000 or 10,000 mg/L (Quimlab Química & Metrologia^®^, Jacareí, Brazil) to obtain the desired concentrations using matrix matching and ultrapure water. The operating conditions of ICP OES were 1200 W of incident power, 12 L/min of plasma gas flow rate, 0.2 L/min of coating gas flow rate, 0.02 L/min of nebulization gas flow rate, 1 bar of nebulizer pressure, 1.0 mL/min of sample introduction flow rate, 1 s of integration time and high resolution. The wavelengths (nm) used were Fe (259.940), K (766.490), Mn (257.610), Na (588.995), P (214.914), Ca (396.847), Zn (213.856), Cu (324.750) and Mg (279.553).

#### 2.3.2. Nitrogen Content

The nitrogen analysis was performed according to Kjeldahl method [22].

### 2.4. Black Beans Chemical Analysis

#### 2.4.1. Pesticides Residues

The pesticide residues analysis was carried out according to QuEChERS method, with adaptations [23]. Aliquots of 5.0 g of black beans samples were weighed in 50 mL disposable screw-capped polypropylene centrifuge bottles. As black beans are dry samples, ultrapure water (approximately 4.5 mL) was added and homogenized. For extraction, acetonitrile (10 mL) containing 1% acetic acid was used. After vortex homogenization, a solid mixture of 6.0 g of magnesium sulfate and 1.5 g of sodium acetate were added. The samples were homogenized again and centrifuged at 3000 rpm for 7 min at 20 °C. Two fortifications were carried out at the first level of quantification (8 μg/kg) for the evaluated pesticides, and method recoveries were calculated considering adequate recovery values ranging from 70 to 120%. Stock solutions from 100 to 400 μg/mL were prepared. Intermediate solutions were made up to the concentrations of the analytical curves, which ranged from 0.002 to 0.008 μg/mL, where 0.002 μg/mL (8.0 μg/kg in the matrix) was the limit of quantification.

The chromatograph was equipped with a binary pump system, automatic injector, degasser and oven column. The column used for the chromatographic separations was a reverse phase ACQUITY UPLC™ BEH C18 (1.7 μm, 2.1 mm × 100 mm) (Waters, Milford, MA, USA). The pre-column was a VanGuard™ BEH C18 (1.7 μm) (Waters, Milford, MA, USA). The mobile phase was as follows: 5 mM ammonium formate, 0.1% formic acid and 10% methanol in deionized water (eluent A) and methanol (eluent B). The initial elution gradient was 82.5% of eluent A, with a linear ramp until reaching 5.5% of the same eluent in a linear curve. The mobile phase flow rate was set at 0.3 mL/min and the injection volume was of 5 μL. The total analysis time was 25 min.

The sequential mass detector was equipped with an electrospray ionization source (Z-Spray^TM^, Waters, Milford, MA, USA) operating in positive ionization mode and a MassLynx^TM^ Version 4.1 workstation. The mass spectrometer operation conditions were optimized for multiple reaction monitoring (MRM) mode, through the indication of precursor ions and product ions. Two ion transitions were selected (*m*/*z*) for each pesticide. The source parameters were capillary voltage 0.98 kV, desolvation temperature of 400 °C and source temperature of 100 °C. Nitrogen was used as the cone gas at flows of 50 L/h. Argon was used as the collision gas at a pressure of 3.5 μbar.

#### 2.4.2. Proximate Composition

Moisture, protein, lipid, dietary fiber and ash contents of beans were determined in triplicate, according to official methods [22]. Carbohydrate content was determined by difference.

#### 2.4.3. Phytate Content

Phytate content was determined in triplicate. Phytates were extracted with HCl 2.4% [24] and sample cleanup was performed using an AG^®^ 1-X8 anion exchange column [25]. Extracts were mixed with Wade’s reagent (0.003 g/L FeCl_3_. 6H_2_O and 0.03 g/L sulfosalicylic acid in distilled water) and absorbance was measured at 500 nm (Shimadzu UV-1800, Kyoto, Japan) [24]. Quantification was performed by external calibration.

#### 2.4.4. Mineral Composition

Bean digestion was performed in triplicate according to official methods [22]. Mineral contents were performed using the same conditions of the soil analysis, described in Section 2.3.1.

#### 2.4.5. Phenolic Compounds

Extraction of soluble and insoluble phenolic compounds from black beans was performed in triplicate, according to an adaptation of the methodology of Matilla and Kumpulainen [26].

For soluble phenolic compounds, 2 grams of sample were vortexed with 20 mL of cold ethanol:water:hydrochloric acid (79:20:1, *v*/*v*/*v*) for 10 min and centrifuged (2500× *g*, 5 min, 10 °C). The supernatant was collected, and the residue re-extracted using the same procedure. Supernatants were combined, the solvent was removed, and the dry residue was reconstituted in HCl 0.01 M.

For insoluble phenolic compounds, we performed sequential alkaline and acid hydrolysis. For the alkaline hydrolysis, the solid residue remaining after the soluble phenolic extraction was incubated with 12 mL of water and 5 mL of NaOH (10 M) at room temperature in the dark for 16 h with orbital agitation (360 rpm). After this period, the pH was adjusted to 2 and the mixture was extracted for 30 s with 15 mL of ethyl acetate. After centrifugation (2500× *g*, 5 min, 10 °C), the supernatant was collected and the extraction procedure repeated twice. Supernatants were combined, the solvent was removed, and the dry residue was reconstituted in methanol 80%. For the acid hydrolysis, the solid residue remaining after the alkaline hydrolysis was incubated with 2.5 mL of concentrated HCl at 85 °C for 30 min. Then, the same extraction with ethyl acetate described for alkaline hydrolysis was performed.

All extracts were filtered through a 0.45 µm cellulose ester membrane (Millipore^®^, São Paulo, Brazil) prior to HPLC analysis.

Phenolic compounds were analyzed according to Inada et al. [27] in an HPLC system (Shimadzu, Kyoto, Japan) composed of LC-20AT quaternary pump, SPD-M20A diode array detector (DAD), control system CBM-20A, DGU-20A5 degasser and SIL-20AC automatic injector coupled to LCMS-2020 mass spectrometer.

Chromatographic separation of non-anthocyanin phenolic compounds was achieved using a reverse phase column (C18, 5 µm, 250 mm × 4.6 mm, Kinetex^®^, Torrance, CA, USA) and the mobile phase consisted of a gradient of 0.3% aqueous formic acid (eluent A) and methanol (eluent B), both containing 1% of acetonitrile. The flow was 1.0 mL/min. Prior to injection, the column was equilibrated with 18% B. After injection, solvent composition was modified to 20% B in 1 min, 43% B in 18 min, and 85% B in 23 min, and kept constant for 30 min. Between injections, 10 min intervals were allowed to re-equilibrate the column with 18% B. Compounds were monitored at DAD from 190 to 370 nm and at mass spectrometer (MS) by negative selected ion monitoring (SIM) mode.

Chromatographic separation of anthocyanins was achieved using a reverse phase column (C18, 5 µm, 150 mm × 4.6 mm, Kinetex^®^) and the mobile phase consisted of a gradient of 1% aqueous formic acid (eluent A) and methanol (eluent B), both containing 2% of acetonitrile. The flow was 1.0 mL/min. Prior to injection, the column was equilibrated with 18% B. After injection, solvent composition was kept constant for 2 min, and then modified to 32% B in 6 min, 52% B in 8 min, and 18% B in 18 min. Between injections, 10 min intervals were allowed to re-equilibrate the column with 18% B. Anthocyanins were monitored at DAD at 530 nm and at MS by positive SIM mode.

Identification of all phenolic compounds was performed by comparison with retention time and UV-Vis absorption and MS spectra of the respective standard. Quantification was performed by external calibration. Identification of compounds for which no commercial standard was available (quercetin-3-*O*-glucoside, myricetin-3-*O*-glucoside, kaempferol-3-*O*-glucoside, malvidin-3-*O*-glucoside and petunidin-3-*O*-glucoside) was performed by MS spectra and, for their quantification, the corresponding aglycone was employed. Pelargonidin and malvidin were quantified together, since their chromatographic separation was not possible.

### 2.5. Statistical Analyses

Data were expressed as mean ± standard deviation. The chemical composition between nonorganic and organic black bean and soil samples from the same region were compared by an unpaired *t*-test. Differences between chemical composition of black bean samples grouped according to the production system (nonorganic or organic), independently of production region, were evaluated by paired *t*-test. All statistical analyses were performed using GraphPad Prism version 7.0 (GraphPad Software, San Diego, CA, USA). Results were considered significant when *p* < 0.05.

## 3. Results and Discussion

### 3.1. Pesticide Residues Were Not Detected in Any Sample

Considering the limit of quantification (8.0 μg/kg), no residues of 294 evaluated pesticides (Table S1) [28] were detected in the samples. However, nine pesticides already reported in Brazilian beans (procymidone, fenpropatrin, permethrin, fipronil, endosulfan, allethrin, phenotrothione, cypermethrin), as well as glyphosate, which is the most employed pesticide worldwide and in Brazil [29], were not evaluated in this study due to analytical limitations.

### 3.2. Organically Produced Black Beans Contain Approximately 20% More Proteins than Nonorganically Produced Ones 

Ashes, lipids, proteins, dietary fibers and carbohydrates contents in all black bean samples were in accordance with the literature [30]. Although lipid contents in organic black beans were, on average, 17% lower than that of nonorganic beans (Table 1), this result is of low significance, as beans are not relevant dietary sources of this nutrient. Organic beans cultivated in the coastal region showed 31% higher protein contents than nonorganic (Table 1). For the metropolitan region, the same behavior was observed, but of a lower magnitude (8%). To the best of our knowledge, protein contents in organic and nonorganic beans have not been investigated. Organic soybeans also showed higher protein contents compared to nonorganic ones [31]. On the other hand, in a meta-analysis of 343 original articles, which investigated several food groups such as fruits, vegetables, cereals, oil seeds and pulses, and herbs and spices, only organic cereals had lower protein contents compared to nonorganic ones, which may be associated with their lower nitrogen input and availability [16].

Soils from organic management farms showed 78% and 55% lower nitrogen contents than that of nonorganic, at the costal and metropolitan regions, respectively (Table 2). This result may be explained by the frequent use of chemical fertilizers (usually rich in nitrogen) at nonorganic farms [16]. At first glance, these data would contradict the protein results observed for our black bean samples. However, it is known that the main pathway for nitrogen absorption in legumes, such as beans and soybeans, is not nitrogen uptake from the soil, but, rather, fixation of atmospheric nitrogen through symbiosis with rhizobium bacteria [32]. In that sense, the use of pesticides may affect this symbiosis by decreasing bacterial population and/or symbiotic efficiency [33], therefore affecting nitrogen fixation and, ultimately, protein legume contents. In this way, glyphosate appears to inhibit aromatic amino acid biosynthesis through inhibition of the enzyme enolpyruvylshikimate-3-phosphate synthase of the shikimate pathway [34]. In our study, glyphosate, the most commonly used herbicide in Brazil has not been evaluated, and and information about soil fertilization practices has not been collected. Therefore, it was not possible to know whether the differences in protein contents were related to the use of this pesticide. However, a meta-analysis with 56 studies shows that, overall, organic farming enhances total microbial abundance and activity in agricultural soils on a global scale. This meta-analysis found that soils from organic systems had 51% higher microbial nitrogen than conventionally farmed ones [35]. We hypothesize that this may partly explain the higher protein contents in organic beans, despite the lower nitrogen contents in the soils from organic management farms observed in our study. Besides that, microbial inoculants can be used to supply the plant’s nitrogen demand, thus increasing grain yield and improving soil fertility [36]. The application of inoculant was reported by the coastal organic producer of our study.

Considering that the consumption of beans represents one of the main sources of dietary protein, especially by low-income people in developing countries [7], access to organic beans may be beneficial to this population.

### 3.3. Lower Phytate Contents in Organic Black Beans May Increase Mineral Bioavailability

In general, the mineral profile of our black bean samples was similar to that reported in the literature [30] (Table 1). Common beans are recognized as an important dietary source of iron, zinc, magnesium, copper, potassium and phosphorous. Organic black beans showed, on average, 35% and 37% lower copper and manganese contents, respectively, than nonorganic ones in both regions. The organic sample from the metropolitan region showed a 7% higher potassium content than the nonorganic one. There were no differences in magnesium and iron contents between organic and nonorganic beans in both regions, despite the variations observed in the soil (Table 2). In general, the contents of all minerals in beans (Table 1) and soils (Table 2) were not correlated, suggesting that plant intrinsic (genetic aspects and carbohydrates contents) and extrinsic factors (weather and soil physical–chemical aspects) influenced mineral acquisition capacity [32].

Although several systematic reviews have been published regarding differences in mineral profiles between organic and nonorganic foods, there is no consensus in the literature. Dangour et al. [15] reported no differences between organic and nonorganic foods in terms of magnesium, calcium, potassium, zinc and copper, except for phosphorus, which showed higher contents in organic foods. Hunter et al. [37] reported the same result regarding higher phosphorus contents in organic plant foods, and for all analyzed minerals in general. Hattab, Bougattass, Hassine and Dridi-Al-Mohandes [38] observed higher levels of minerals in organic tomatoes, lettuces and strawberries when compared to nonorganic. Barański et al. [16] reported lower manganese contents and slightly higher zinc and magnesium contents in organic crops compared to nonorganic ones, while calcium, copper and iron showed no significant differences. Worthington [39] concluded that organic foods had higher iron, magnesium and phosphorus levels than nonorganic ones, possibly due to a stronger association of plants and microorganisms in organic soils.

Phytate is an antinutritional factor that chelates divalent cations, such as calcium, zinc, magnesium and iron, decreasing their bioavailability [40]. The mean phytate content in black beans samples (Table 1) was similar to that reported by other studies [41]. In both regions, organic samples presented, on average, 10% lower phytate contents in comparison to nonorganic beans. Phytate is the main storage form of phosphate and inositol in seeds, grains and nuts [40], and the use of synthetic fertilizers rich in phosphorus may increase phytate content [42]. In fact, we observed higher phosphorus content in soils from nonorganic farms in comparison to organic ones. Even though organic and nonorganic black beans showed similar iron contents, we may suppose that iron bioavailability in organic beans would be higher than in nonorganic ones. This could be especially relevant for low-income populations and vegetarians, groups vulnerable to iron deficiency and for whom beans represent an important dietary source of this mineral [7].

### 3.4. Organic Black Beans Present 28% Higher Phenolic Compounds Contents than Nonorganic Ones

**Figure 2:**
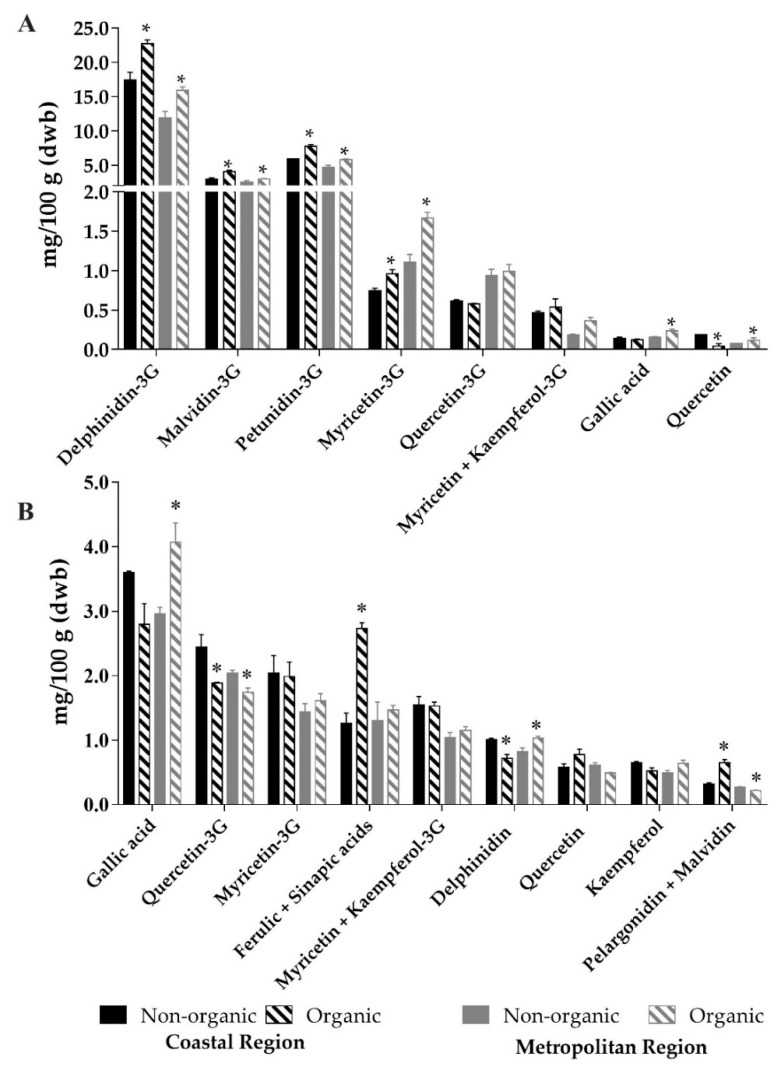
Contents (mg/100 g) of soluble (**A**) and insoluble phenolic compounds (**B**) in black beans cultivated at the coastal and metropolitan regions. Asterisks indicate significant differences between nonorganic and organic samples from the same region (unpaired *t*-test, *p* < 0.05). G = glucoside.

The phenolic compounds profile was similar in the four black bean samples. These compounds were mainly found in the soluble fraction, which corresponded, on average, to 69% of the total phenolic contents. Nine compounds were found as soluble phenolics: delphinidin-3-*O*-glucoside, petunidin-3-*O*-glucoside, malvidin-3-*O*-glucoside, myricetin-3-*O*-glucoside, quercetin-3-*O*-glucoside, kaempferol-3-*O*-glucoside, myricetin, gallic acid and quercetin. This profile has already been reported for black beans by other authors [13,43]. Anthocyanins were the most abundant, corresponding to 93%, on average, of total soluble phenolic compounds (Figure 2A).

Within samples from the same region, organic beans showed 25% higher contents of soluble phenolic compounds, mostly anthocyanins, than nonorganic ones. According to a meta-analysis based on 343 peer-reviewed papers [16], organic crops show higher phenolic compounds contents, especially anthocyanins, when compared to nonorganic. It is worth noting that none of the papers investigated in this meta-analysis have studied beans, which were, to the best of our knowledge, investigated for the first time in this work regarding differences between organic and nonorganic crops.

Twelve phenolic compounds were found in the insoluble fraction. In comparison to those of the soluble fraction, no anthocyanins glucosides were identified, but three anthocyanidins (aglycones) were observed: delphinidin, malvidin and pelargonidin. Moreover, this fraction also contained two other phenolic acids (ferulic and sinapic acids) and one flavonol (kaempferol) that were not present in the soluble fraction (Figure 2B). Luthria and Pastor-Corrales [44] found ferulic, sinapic and *p*-coumaric acids in the insoluble fraction of black beans, obtained after alkaline and acid hydrolysis, but not in the soluble fraction. Ranilla, Genovese and Lajolo [45] reported that phenolic acids are mainly present in the cotyledon of black beans, whereas a predominance of flavonoids is observed in the seed coat. In the insoluble fraction, differences between organic and nonorganic samples were less frequent and less consistent than those observed in the soluble fraction. Considering that soluble phenolics are mainly found in the seed coat of legumes [45], one could hypothesize that these would be more susceptible to the influence of the production system than those insoluble forms found in the inner part of the seed.

**Figure 3:**
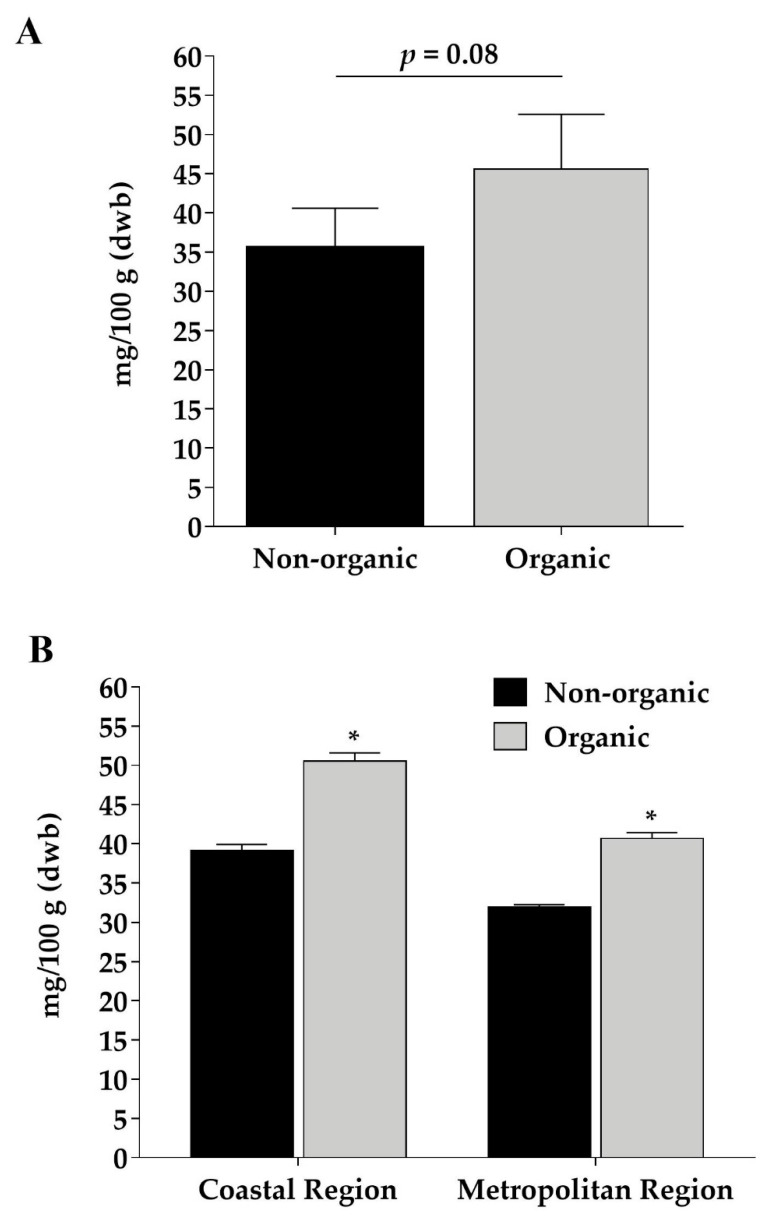
Differences in contents (mg/100 g) of total phenolic compounds (sum of soluble + insoluble contents, which were analyzed by High Performance Liquid Chromatography) of organic and nonorganic black beans, independently of the harvest region (paired *t*-test) (**A**) and according to coastal and metropolitan regions (unpaired *t*-test) (**B**). Asterisks indicate differences at 95% confidence.

When grouping samples according to agricultural practice, regardless of the region, we observed that organic beans showed a tendency (*p* = 0.08) to present a higher total content of phenolic compounds (45.6 ± 6.9 mg/100 g) when compared to nonorganic beans (35.5 ± 5.1 mg/100 g) (Figure 3A). When coastal and metropolitan regions were considered separately, organic beans showed, on average, 28% higher total phenolic compounds contents than nonorganic ones (Figure 3B). This difference was caused by higher soluble phenolics contents in the organic samples (32.5 mg/100 g, on average) compared to the nonorganic samples (24.2 mg/100 g, on average), but not by differences in insoluble phenolics (13.2 and 12.2 mg/100 g, respectively). Giusti et al. [46] observed higher phenolic acid contents for organic legumes compared to conventional ones. On the other hand, Jakopic et al. [47] did not find differences in total phenolic compounds between organic and nonorganic dwarf French beans, although a higher content of catechin was observed in the organic sample.

Despite the limited number of samples analyzed, our results strongly suggest a positive influence of organic farming on black beans phenolic compounds. Barański et al. [16] reported that organic fruits and vegetables have higher concentrations of phenolic compounds than nonorganic crops. Although some pesticides were not investigated in our study due to methodological limitations, if one assumes that the farms were following organic certification rules, and, therefore, pesticides were absent, organic plants may have been more susceptible to biotic stress, such as pest attacks or diseases, and/or abiotic stress, such as physical damage and water or nutrient scarcity [17,34]. The higher contents of phenolic compounds in organic food samples may be associated with responses to these stresses suffered by the plant in organic agricultural practice when compared to nonorganic. In that scenario, plant secondary metabolism may be affected, leading to an increased production of phenolic compounds and other substances for their protection. In addition to environmental stress, organic management seems to be associated with plant oxidative stress. Oliveira et al. [48] observed higher lipid peroxidation, superoxide dismutase activity, soluble solids, vitamin C and phenolic compounds in organic tomatoes when compared to nonorganic tomatoes. Furthermore, some pesticides may decrease secondary metabolite synthesis by inhibiting the shikimic acid pathway that is part of phenolic compounds’ biosynthesis [17,34].

The major drawback of this work is the limited number of black bean samples. Nevertheless, these samples allow the association between the chemical composition and the production system, as climatic confounding factors (e.g., weather conditions, altitude, sun exposure) were carefully controlled by the study design (samples grown at geographically near farms and from close harvest periods). Soil composition, which is another confounding factor, was analyzed and taken into account when comparing production systems. Most studies in the literature do not have such control over sampling, and thus require larger sample sizes and often do not observe differences in chemical composition associated with the production system. Barański et al. [16] highlights that studies comparing the impacts of agronomic practices on crop/food composition should minimize sample heterogeneity.

## 4. Conclusions

In conclusion, organic farming improved the nutritional profile (20% more proteins, 10% less phytate) and increased phenolic compounds contents (28%) of black beans. In this sense, it could be interesting to encourage organic farming, especially in developing countries in which beans are a staple food. Nevertheless, further studies with wider sampling must be carried out to confirm the impact of organic agriculture on the chemical composition of beans.

## Figures and Tables

**Table 1:** Proximate composition, phytate and minerals of nonorganic and organic black beans cultivated at the coastal and metropolitan regions.

	Coastal Region		Metropolitan Region	
	Nonorganic	Organic	Nonorganic	Organic
	Proximate composition and phytate (g/100 g, dry weight basis)			
Lipid	1.7 ± 0.1	1.4 ± 0.0 *	1.9 ± 0.1	1.6 ± 0.1 *
Protein	19.2 ± 0.4	25.2 ± 0.1 *	22.4 ± 0.0	24.3 ± 0.3 *
Ash	4.2 ± 0.0	3.8 ± 0.2 *	4.4 ± 0.2	4.4 ± 0.1
Carbohydrate	26.8	27.7	25.8	23.1
Total dietary fiber	35.5 ± 2.6	30.8 ± 1.2 *	31.2 ± 0.5	32.1 ± 0.9
Insoluble dietary fiber	31.9 ± 1.4	29.8 ± 0.5 *	27.2 ± 0.7	27.0 ± 0.8
Soluble dietary fiber	3.60	1.00	3.95	5.11
Phytate	1.86 ± 0.02	1.76 ± 0.02 *	2.08 ± 0.07	1.83 ± 0.11 *
	Minerals (mg/100 g, dry weight basis)			
Ca	158.6 ± 3.2	102.2 ± 11.4 *	152.9 ± 2.4	176.4 ± 3.4 *
Cu	1.01 ± 0.08	0.58 ± 0.05 *	1.15 ± 0.03	0.83 ± 0.01 *
Fe	4.13 ± 0.2	4.11 ± 0.4	4.56 ± 0.2	4.69 ± 0.3
K	1351.4 ± 25.6	1354.9 ± 3.8	1368.7 ± 11.7	1461.6 ± 21.5 *
Mg	156.4 ± 4.3	146.2 ± 3.1	171.1 ± 2.6	169.1 ± 2.4
Mn	1.97 ± 0.15	1.01 ± 0.16 *	1.46 ± 0.02	1.10 ± 0.08 *
Na	15.1 ± 9.5	10.6 ± 4.1	20.9 ± 0.17	8.98 ± 2.4 *
P	249.0 ± 56.4	318.2 ± 51.4	394.5 ± 14.4	372.9 ± 27.9
Zn	1.95 ± 0.10	1.61 ± 0.27	2.57 ± 0.08	2.31 ± 0.04 *

Results were expressed as mean ± standard deviation (n = 3). The asterisk indicates significant difference between nonorganic and organic black beans samples from the same region (unpaired *t*-test, *p* < 0.05).

**Table 2:** Soil mineral composition (mg/kg, dry weight basis) of nonorganic and organic management farms located at the coastal and metropolitan regions.

	Coastal Region		Metropolitan Region	
	Nonorganic	Organic	Nonorganic	Organic
**Ca**	938.6 ± 1.8	131.8 ± 55.6 *	941.5 ± 42.4	1322 ± 223
**Cu**	9.4 ± 0.07	traces *	8.75 ± 0.3	0.69 ± 0.47 *
**Fe**	18,356 ± 725	1228 ± 239 *	3625 ± 80	24,351 ± 2104 *
**K**	2062 ± 57	traces *	184.4 ± 14.3	1306 ± 150 *
**Mg**	3171 ± 45	traces *	61.1 ± 18	3311 ± 361 *
**Mn**	219.7 ± 3.7	11.7 ± 1.9 *	8.82 ± 2.9	275.4 ± 33.9 *
**Na**	traces	traces	traces	traces
**P**	695.9 ± 2.6	81.5 ± 10.5 *	938.4 ± 20.6	267.3 ± 35.1 *
**Zn**	45.6 ± 1.0	traces *	12.46 ± 1.2	34.1 ± 5.2 *
**N**	4801.1 ± 42.7	1048.2 ± 24.3 *	6087.2 ± 199.1	2701.8 ± 14.9 *

Results were expressed as mean ± standard deviation (n = 3). The asterisk indicates significant difference between soil samples of nonorganic and organic management farms from the same region (unpaired *t*-test, *p* < 0.05). “Traces” means that values were below the limits of quantification and above the limits of detection of the analytical method for each mineral.

## Data Availability

The data presented in this study are available on request from the corresponding author.

## Supplementary Materials

The following are available online at https://www.mdpi.com/article/10.3390/foods10040900/s1, Table S1: List of pesticides analyzed by LC-MS-MS and their respective chemical classes.
