# Supplementary material for: Organic Black Beans (Phaseolus vulgaris L.) from Rio de Janeiro State, Brazil, Present More Phenolic Compounds and Better Nutritional Profile Than Nonorganic
Source: Foods. 2021 Apr 19;10(4):900. doi: 10.3390/foods10040900 (PMC8073876; doi:10.3390/foods10040900)
Supplement: Supplementary file 1 [file foods-10-00900-s001.zip › foods-1182937-supplementary.pdf]

Table S1. List of pesticides analyzed by LC-MS-MS and their respective chemical classes [1]

**Chemical Class**	**Pesticide**
Acetamide	cymoxanil
Aliphatic unsaturated ester	methoprene
Amide	dichlofluanid, DMSA, DMST, isocarbamide, tolylfluanid
Anilide	carboxin, fenhexamid, methfuroxam, monalide, oxycarboxin, propanil
Azoles	amicarbazone, azaconazol, bitertanol, bromuconazole, carfentrazone-ethyl, clothianidin, cyazofamid, cyproconazole, diniconazole, difenoconazole, epoxiconazole, ethiprole, etoxazole, fenbuconazole, fenpyroximate, fipronil, flusilazole, flutriafol, fluquinconazole, fluxapyroxad, hexaconazole, imazalil, imazaquin, imibenconazole, isoxaflutole, metconazole, myclobutanil, oxadiargyl, paclobutrazol, penconazole, prochloraz, propiconazole, sulfentrazone, tebuconazole, tebufenpyrad, tetraconazole, thiabendazole, triadimefon, triadimenol, triflumizol, triticonazole, uniconazole
Benzamide	2,6-dichlorobenzamide, flutolanil
Benzimidazole	fuberidazole
Benzophenone	metrafenone
Benzofuran	ethofumesate
Benzonitrile	ioxynil
Butenolide	spiromesifen
Carbamate	3-hidroxycarbofuran, alanycarb, aldicarb, aldicarb sulfone, aldicarb sulfoxide, aminocarb, bendiocarb, benfuracarb, bifenazate, butocarboxim, butocarboxim sulfoxide, carbaryl, carbendazim, carbetamide, carbofuran, carbosulfan,

	desmedipham, diethofencarb, dioxacarbe, EPTC, esprocarbe, ethiofencarb, ethiofencarbe sulfone, ethiofencarbe sulfoxide, fenobucarb, fenoxycarb, furathiocarb, iprovalicarb, isoprocarbe, karbutilate, methiocarb, methiocarb sulfone, methiocarbe sulfoxide, methomyl, molinate, oxamyl, oxamyl oxime, phenmedipham, pirimicarb, pirimicarb desmethyl, propamocarb, propham, propoxur, thiobencarb, thiodicarb,thiofanox, tiofanox sulfone, tiofanox sulfoxide, thiophanate-methyl
Carboxamide	benalaxyl, carpropamid, chlorantraniliprole, cyflufenamid, etobenzanid, flubendiamide, mepronil, metalaxyl, metalaxyl m, mandipropamid, propyzamide, zoxamide
Chloroacetamide	alachlor, butachlor, dimethenamid
Cyclohexanedione	clethodim cycloxydim, tepraloxydim
Dinitroaniline	pendimethalin
Dioxoles	piperonyl butoxide
Dithiolane	isoprothiolane
Flavonoid	rotenone
Hydrazine	halofenozide, methoxyfenozide, tebufenozide
Imidazolinone	imazethapyr, imazapic
Imine	fluthiacet-methyl
Lactone	abamectin, doramectin, emamectin benzoate, eprinomectin, moxidectin, spinetoram, spinosad, spirodiclofen
Morpholine	dimethomorph, dodemorph, fenpropimorph, spiroxamine, tridemorph
Neonicotinoid	acetamiprid, imidacloprid, thiamethoxam
Organophosphorus	acephate, azamethiphos, azinphos-ethyl, azinphos-methyl, cadusafos, chlorpyrifos, chlorpyrifos methyl,

	chlorfenvinphos, coumaphos, demeton-s-methyl, diazinon, dichlorvos, dicrotophos, dimethoate, disulfoton, etrimfos, ethion, ethoprophos, fenamiphos, fenthion, fenthion sulfoxide, heptenophos, isocarbophos, isofenphos, isoxathion, malathion, methidathion, mephosfolan, methamidophos, mevinphos, monocrotophós, omethoate, phosphamidon, pirimiphos-ethyl, pirimiphos-methyl, phenthoate, phosalone, phosmet, phoxim, profenophos, pyrazophos, pyridaphention, quinalphos, temephos, terbufos, tolclofos-methyl, triazophos, trichlorphon, vamidothion, vamidothion sulfone
Organotin	cyhexatin
Oxadiazine	indoxacarb
Oxazolidine	famoxadone, oxadixyl
Oxyacetamide	flufenacet, mefenacet
Phenyl Ether	lactofen
Piperazine	triforine
Piperidine	fenpropidin
Propionate	quizalofop-ethyl
Pyridazine	pyridaben, norflurazon
Pyridine	boscalid, flonicamid, fluazifop-p-butyl ether, nitenpyram, pyriproxyfen, pyrifenox, thiacloprid
Pyrimidine	bupirimate, cyprodinil, ethirimol, fenarimol, mepanipirym, nuarimol, pyrimethanil
Quinazoline	fenazaquim, proquinazid
Quinoline	quinoxyfen
Strobilurin	azoxystrobin, dimoxystrobin, fenamidone, fluoxastrobin, kresoxim-methyl, picoxystrobina, pyraclostrobin,

	trifloxystrobin
Sulfonamide	flusulfamide
Sulphite ester	propargite
Terpene	azadirachtin, ethofenprox
Tetrazine	clofentezine
Thiadiazine	buprofezin
Thiazole	acibenzolar-s-methyl, hexytiazox, tricyclazole
Triazine	ametryne, atrazine, cyromazine, methoprotryne, metribuzin, prometryn, prometon, propazine, pymetrozine, sebuthylazine, simazine, simetryn, terbumeton, terbutryn
Triketone	mesotrione
Urea	daimuron, diafenthiuron, diflubenzuron, diuron, chloroxuron, chlorbromuron, chlorfluazuron, chlorimuron-ethyl, cumyluron, ethidimuron, fenuron, flufenoxuron, forchlorfenuron, imazosulfuron, isoproturon, linuron, metobromuron, metoxuron, monolinuron, metsulfuron methyl, neburon, novaluron, pencycuron, siduron, tebuthiuron, teflubenzuron, triflumuron, triflusulfuron-methyl
Xylylalanine	furalaxyl

[1] Chemical class of pesticides according to PubChem database and EURL DataPool website (https://www.eurl-pesticides-datapool.eu) [28].
